# Absence of a relationship between immunophenotypic and colony enumeration analysis of endothelial progenitor cells in clinical haematopoietic cell sources

**DOI:** 10.1186/1479-5876-5-37

**Published:** 2007-07-18

**Authors:** Olga Tura, G Robin Barclay, Huw Roddie, John Davies, Marc L Turner

**Affiliations:** 1 SNBTS Adult Cell Therapy Group, Scottish Centre for Regenerative Medicine, University of Edinburgh School of Clinical Sciences, The Chancellor's Building, 49 Little France Crescent, Edinburgh, EH16 4SB, UK; 2 NHS Lothian University Hospitals Division, Department of Haematology, Western General Hospital, Edinburgh, EH4 2XU, UK

## Abstract

**Background:**

The discovery of adult endothelial progenitor cells (EPC) offers potential for vascular regenerative therapies. The expression of CD34 and VEGFR2 by EPC indicates a close relationship with haematopoietic progenitor cells (HPC), and HPC-rich sources have been used to treat cardiac and limb ischaemias with apparent clinical benefit. However, the laboratory characterisation of the vasculogenic capability of potential or actual therapeutic cell autograft sources is uncertain since the description of EPC remains elusive. Various definitions of EPC based on phenotype and more recently on colony formation (CFU-EPC) have been proposed.

**Methods:**

We determined EPC as defined by proposed phenotype definitions (flow cytometry) and by CFU-EPC in HPC-rich sources: bone marrow (BM); cord blood (CB); and G-CSF-mobilised peripheral blood (mPB), and in HPC-poor normal peripheral blood (nPB).

**Results:**

As expected, the highest numbers of cells expressing the HPC markers CD34 or CD133 were found in mPB and least in nPB. The proportions of CD34^+ ^cells co-expressing CD133 is of the order mPB>CB>BM≈nPB. CD34^+ ^cells co-expressing VEGFR2 were also most frequent in mPB. In contrast, CFU-EPC were virtually absent in mPB and were most readily detected in nPB, the source lowest in HPC.

**Conclusion:**

HPC sources differ in their content of putative EPC. Normal peripheral blood, poor in HPC and in HPC-related phenotypically defined EPC, is the richest source of CFU-EPC, suggesting no direct relationship between the proposed EPC immunophenotypes and CFU-EPC potential. It is not apparent whether either of these EPC measurements, or any, is an appropriate indicator of the therapeutic vasculogenic potential of autologous HSC sources.

## Background

Until recently postnatal vascular repair and regeneration was thought to result exclusively from angiogenesis, the outgrowth of fully differentiated mature endothelial cells (EC) from pre-existing blood vessels. The discovery that mononuclear cells in peripheral blood have the potential to differentiate into endothelial cells and may give rise to de novo vasculogenesis [1-3], a process hitherto thought only to occur in the developing embryo, has stimulated growing interest in clinical use of locally injected autologous cells to promote revascularisation of ischaemic tissue. These circulating adult endothelial progenitor cells (EPC) appear to share a common precursor with haematopoietic progenitor cells (HPC) which was first recognised in early embryogenesis and termed the haemangioblast [4-6].

HPC are routinely employed in clinical haematopoietic reconstitution, commonly following myeloablative chemotherapy for leukaemias when they are harvested for autologous use during periods of remission. HPC for clinical use were harvested initially from bone marrow, but latterly are predominantly harvested by apheresis following mobilisation of HPC from bone marrow to peripheral blood (PBHPC) following administration of G-CSF. They may also be harvested from allogeneic donors from the same sources. Haematopoiesis is recognised to depend upon the HPC dose administered for rapid and sustained engraftment, and the haematopoietic potential of the HPC graft is determined by the numbers of CD34-expressing mononuclear cells [7] or by expression of CD133 which may identify an earlier more pluripotent HPC population [8-10].

EPC have much in common with HPC and share the phenotypic markers CD34 and CD133. Thus they may be regarded as a subset of the CD34^+ ^or CD133^+ ^mononuclear cells found in bone marrow and blood, and cells co-expressing either of these markers together with VEGF-receptor-2 (VEGFR2, also known as KDR) have been ascribed endothelial progenitor activity [2,11-14]. However the ontogenic relationship between expression of these markers and maturity, pluripotency and function is not clear [9,10,15,16]. It is reported that VEGFR2 may be co-expressed by subsets of cells expressing CD133 or CD34 exclusively or jointly, and while VEGFR2 expression has been associated with EPC activity it is not clear whether all or any phenotypically definable subset of these VEGFR2 expressing cells are exclusively vasculogenic or whether they retain haematopoietic potential [11,17-19]. Further, it appears that some circulating mononuclear cells with EPC potential may not clearly express either CD133 or CD34 [20-22]. It now appears probable that the EPC, like other tissue stem and progenitor cells, are not a distinct entity with a unique definable phenotype but a continuum along a range of development [23-26], the root of which is shared with haematopoietic stem cells. Currently, many groups use their own often relatively exclusive EPC definitions based on their experimental and/or clinical outcomes, but usually combining one, two or three of the markers CD34; CD133; VEGFR2. This makes it very difficult to interpret and compare results between different studies.

In the face of an uncertain phenotype, EPC colony forming unit (CFU) assays have emerged as alternative specific enumeration system for EPC [21-23]. The CFU assay proposed by Hill et al[27] determines early or spontaneous CFU-EPC formation based on mononuclear cell colony outgrowth over 5 days on fibronectin coated plates in simple medium without growth factors. Characteristic colonies are evident even at low frequencies which would be difficult to determine statistically by flow cytometry if the phenotype of the colony forming cell were known. This colony assay is now available as a standardized commercial kit (see methods) which should allow inter-laboratory comparison of results, and has been applied in a number of recent studies of different clinical conditions either alone [27-31] or together with phenotype studies [32-35], to demonstrate increased or reduced numbers of circulating endothelial progenitor cells.

Identification of suitable EPC sources is of the highest importance as a prerequisite for clinical EPC use, and it would appear that because of their interrelationship EPC might be sourced from the HPC sources that have been employed for autologous haematopoietic reconstitution, namely bone marrow or PBHPC. Another HPC-rich source which might be considered as an EPC source is allogeneic umbilical cord blood (CB). However, no clinical studies of allogeneic EPC graft sources have been reported to date since they are likely to require continual immunosuppression to prevent rejection, and the risk versus benefit of that in likely patient groups has not been assessed. While poor in HPC, normal peripheral blood mononuclear cells may contain other useful stem cells. Different groups have preferred most of the different autologous sources or mononuclear cell subpopulations of these as EPC sources for clinical administration. Such studies have generally reported clinical benefit but have not demonstrate any obvious relative advantage or disadvantage of any source over another in clinical vasculogenesis in myocardial or severe limb ischaemia [36-43], including HPC-poor normal peripheral blood mononuclear cells following *ex vivo *conditioning [37].

Since therapeutic EPC would be better sourced from patients themselves, there is concern that some patients requiring vascular regeneration may have numerically or functionally compromised EPC which contributes to their clinical condition, and that they should be assessed for this. It may be that alternate autologous sources or *ex vivo *expansion or maturation might enhance the EPC potential of grafts in such cases. It is also probable that any clinical effect will be dependent on the dose and activity of the effector cells. While clinical vasculogenesis does not yet appear to have an equivalent determinant to CD34 phenotype as used for clinical HPC graft assessment of potency for haematopoietic reconstitution[7], the most prevalent *in vitro *assays to determine EPC number or potency which are emerging in practise are the immunophenotype assay based on combinations of expression of VEGFR2 and CD34 and/or CD133, and the CFU-EPC assay described by Hill et al[27]. We have therefore used these assays to compare putative EPC numbers in potential clinical sources of EPC, namely the HPC-rich autologous sources bone marrow and G-CSF-mobilised peripheral blood, HPC-rich allogeneic umbilical cord blood, and HPC-poor autologous normal peripheral blood.

## Methods

### Collection of samples

Venous blood samples (10 ml) were collected in heparin from healthy blood donors (normal peripheral blood, nPB) and immediately following cell-separator leukapheresis collection of G-CSF mobilised peripheral blood (mPB) from patients for autologous transplant (mPBp) and from healthy donors for allogeneic transplant (mPBd). Cord blood (CB) products (20–50 ml) were aspirated from the umbilical placental veins from normal caesarean deliveries. Bone marrow (BM) samples (3 ml) were obtained by aspiration from the posterior iliac crest of haematologically normal donors. Appropriate ethics committee approval was obtained and written informed consent from each patient.

### Flow cytometry analysis

Cells were phenotyped in the whole blood samples by flow cytometry. Cells were directly stained and analysed for phenotypic expression of surface markers using anti-human monoclonal antibodies (MAbs) conjugated to phycoerythrin (PE), fluorescein isothiocynate (FITC), Peridin Chlorophylla protein (PerCP) or Allophycocyanin (APC). The MAbs used included anti-CD45-PerCP, anti-CD34-FITC, (Becton Dickinson, Oxford, UK), anti-VEGFR2-PE (R&D systems, UK), and anti-CD133-APC (Myltenyi Biotec, UK). Negative controls without antibody were used to establish positive stain boundaries, having first confirmed that tests without antibody did not differ from tests with appropriate isotype non-specific antibody controls in samples stained in whole blood in preliminary experimentation. Samples were stained with none, single, double, triple and quadruple antibodies over the sequence none, CD45, CD34, CD133 and VEGFR2, in a five tube panel. 100 ul of undiluted blood sample was stained with appropriate amounts of antibodies for 30 minutes in the dark; erythrocytes were lysed using lysing solution (Becton Dickinson, UK) for 20 minutes in the dark. Samples were then centrifuged at 200 g for 10 minutes and washed twice with phosphate buffered saline (PBS). The cell pellets were resuspended in Cell Fix solution (Becton Dickinson, UK). For each sample 50,000–100,000 events (of viable cells only), were acquired using a FACSCalibur flow cytometer and CellQuest software (Becton Dickinson, UK). The viable leucocytes were defined by forward versus side-scatter distribution and CD45 expression, which was related to total white count to determine absolute numbers. Other cells were determined as a proportion of total leucocytes from which their absolute numbers could be calculated. CD34, CD133 and VEGFR2 staining was checked with and without CD45 gating to ensure no rare CD45-negative cells expressing these markers were present.

### Colony Forming Units-Endothelial Progenitor Cells (CFU-EPC)

This assay is based on that described by Hill et al[27] but is carried out using commercial kit reagents according to the manufacturers recommendations (CFU-Hill, StemCell Technologies, UK). Mononuclear cells were isolated by buoyant-density centrifugation and resuspended at 2.5 × 10^6 ^cells/ml in Complete Endothelial Culture Medium (CECM) comprising Endocult Basal Medium (StemCell Technologies, UK) supplemented with 1/5 dilution of Endocult supplements (StemCell Technologies, UK) and plated at 2 ml/well in fibronectin-coated 6-well plates (Becton Dickinson, UK) and incubated for two days at 37 C, 5% CO_2 _with 95% humidity. After two days, when mature endothelial cells and monocytes had adhered[27], the non-adherent cells containing EPC were removed, counted and resuspended at 0.5 to 1 × 10^6 ^cells/ml in fresh CECM in fibronectin-coated 24-well plate at 1 ml/well (Becton Dickinson, UK) for a further three days at 37 C, 5% CO_2 _with 95% humidity. The colonies per well were then identified and counted, and the results were expressed as colonies per million non-adherent cells plated. The typical colonies were defined following the published method[27] and StemCell Technologies' technical manual as a central core of "round" cells surrounded by elongated "sprouting" cells at the periphery and are classified as colony forming unit endothelial progenitor cell (CFU-EPC).

### Statistical analysis

The different sets of results were compared using non-parametric tests (Wilcoxon matched pairs test or Mann-Whitney test, depending on whether pairing was possible). GraphPad or NCSS statistical software packages were used.

## Results

### Assessment of CD34^+ ^cells

**Figure 1:**
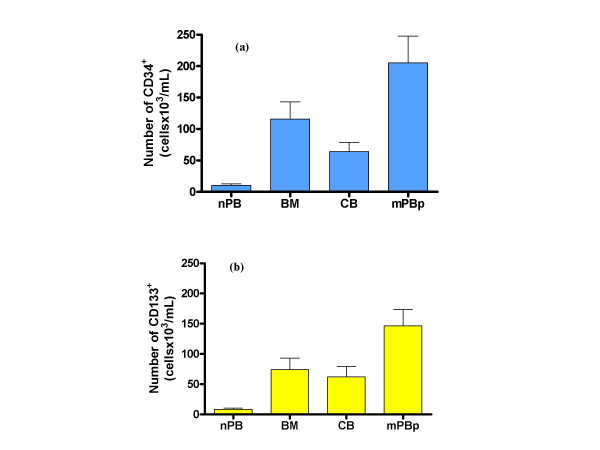
**Expression of CD34 or CD133 markers in haematopoietic stem cell clinical transplant sources and normal blood**. Total numbers of cells expressing CD34 (a) or CD133 (b) in the different sources tested. Sources were normal (non-mobilised) peripheral blood (nPB), bone marrow (BM), umbilical cord blood (CB) and G-CSF-mobilised peripheral blood samples (mPBP) from patients for autologous grafts.

The expression of CD34 on leukocytes was analysed in 10 bone marrow (BM); 21 cord blood (CB); and 27 mobilised peripheral blood samples from patients for autologous transplant (mPBp). The results showed that mPBp had significantly higher numbers of CD34^+ ^than BM and CB (Figure 1a). Normal peripheral blood (not mobilised) (nPB) (n = 16) contained a very low number of CD34^+^ cells compared to the other sources (Figure 1a). Studies were also conducted on mobilised peripheral blood of healthy allogeneic HPC donors (mPBd), but these present at a much lower frequency than patients for autograft. In all cases throughout this study mPBd showed similar results to mPBp, but results are generally not presented here because numbers were too low for meaningful statistical analysis. mPBd results from a larger series will be presented in a subsequent paper which focuses on the effect of G-CSF administration on circulating EPC (manuscript in preparation).

### Assessment of CD133^+ ^cells

The expression of CD133 on leukocytes was analysed in 8 bone marrow (BM), 7 cord blood (CB), and 7 mobilised peripheral blood samples from patients for autologous transplant (mPBp). The distribution of CD133^+ ^cells between sample groups was similar to that of CD34^+ ^cells in that again the highest number of CD133^+ ^cells was seen in mPBp samples (Figure 1b), suggesting that G-CSF administration mobilises CD34^+ ^and CD133^+ ^cells in similar ways. Similarly to CD34^+ ^cells, the numbers of CD133^+ ^cells in normal peripheral blood (nPB: n = 16) were very low compared to the other sources tested (Figure 1b).

### Assessment of CD133 and CD34 co-expression

**Figure 2:**
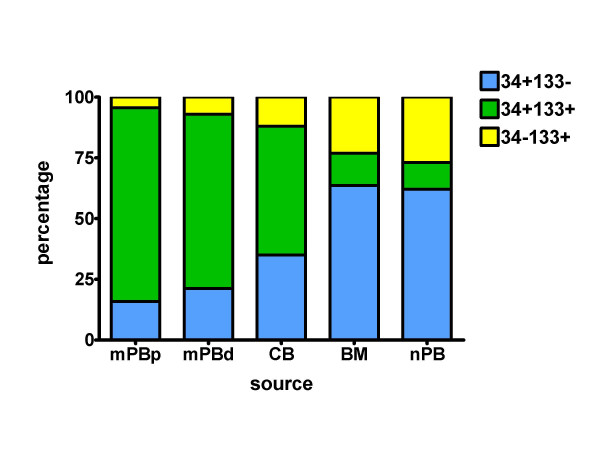
**Relative proportions of CD34+ and/or 133+ cells in different sources**. Percentages (of their summed populations) of CD34^+ ^cells negative for CD133 expression (blue), CD133^+ ^cells negative for CD34 expression (yellow) and cells co-expressing CD133 and CD34 markers (green) were compared between various haematopoietic stem cell clinical transplant sources and normal blood. Normal (non-mobilised) peripheral blood (nPB), bone marrow (BM), umbilical cord blood (CB) and G-CSF-mobilised peripheral blood samples (mPBp, autologous patients; or mPBd, allogeneic donors).

Analysis of CD133 expression by CD34^+ ^cells in each sample group showed that mobilised blood samples (mPB) (79.8%) had markedly higher co-expression of CD133 by CD34^+ ^cells as compared to CB (53%), BM (13%) or nPB (11%) (Table 1). The relative proportions of the CD34^+^CD133^- ^CD34^+^CD133^+ ^and CD34^-^CD133^+ ^subpopulations of cells for each group are shown in Figure 2. In mobilised peripheral blood the jointly-expressing CD34^+^CD133^+ ^cells are the major subpopulation, true for both patients and healthy adult PBHPC donors, whereas in bone marrow and in normal peripheral blood the jointly-expressing cells are a minor subpopulation, outnumbered by both singly-expressing CD133^+^(CD34^-^) and CD34^+^(CD133^-^) populations of which the CD34^+^(CD133^-^) is the major subpopulation. Cord blood shows an intermediate pattern of subpopulation representation.

**Table 1:** Percentage of cells expressing CD34 and/or CD133 in haematopoietic stem cell clinical transplant sources and normal blood.

Sources	BM	CB	mPBP	nPB
total leucocytes/ml (10^6^)	1.63 (0.3) n = 10	1.54 (0.4) n = 21	1.41 (0.26) n = 27	1.17 (0.41) n = 16
CD34+ (% of wbc)	0.71 (0.1) n = 10	0.42 (0.04) n = 21	1.46 (0.36) n = 27	0.09 (0.02) n = 16
CD133+ (% of wbc)	0.46 (0.07) n = 8	0.4 (0.05) n = 7	1.04 (0.2) n = 7	0.07 (0.01) n = 16
CD133+ (% of CD34+)	13 (5.65) n = 8	53 (6.14) n = 7	79.76 (5.5) n = 7	11 (3.96) n = 16

Percentages of cells expressing CD34 and/or CD133 alone or together were compared between the various sources tested. Normal (non-mobilised) peripheral blood (nPB), bone marrow (BM), umbilical cord blood (CB) and G-CSF-mobilised peripheral blood samples (mPBP). Results are expressed as the mean (standard error); n = number of samples tested. White blood cells (wbc) are all leucocytes in the samples analysed, including mononuclear and granulocytic populations.

### Assessment of the total numbers of CD34^+ ^and/or CD133^+ ^cells co-expressing VEGFR2 in the different sources

**Figure 3:**
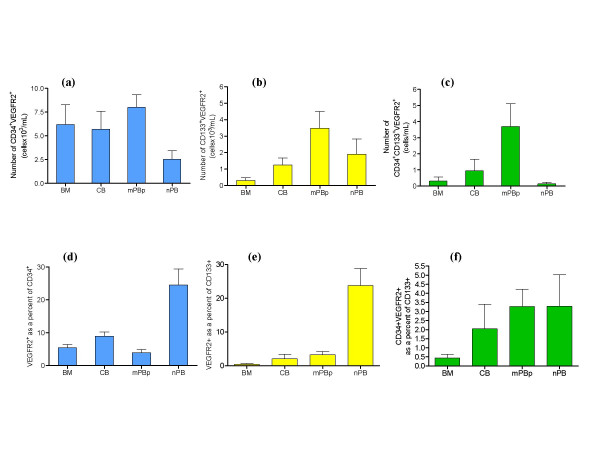
**Number and percentage of putative EPC characterised by different phenotypic definitions in haematopoietic stem cell clinical transplant sources and normal blood**. Number 3a) and percentage 3d) of CD34+ cells co-expressing VEGFR2 in the different sources tested.; Number 3b) and percentage 3e) of CD133+ cells co-expressing VEGFR2 in the different sources tested and Number 3c) and percentage 3f) of CD34^+^CD133^+ ^cells co-expressing VEGFR2 in the different sources tested. Note different Y axis scales between figures.

Mobilised peripheral blood (mPB) was the source with the highest number of CD34^+ ^and/or CD133^+ ^cells co-expressing VEGFR2 compared to the other sources tested (Figure 3a, b, c). The total number of CD34^+ ^and/or CD133^+ ^cells co-expressing VEGFR2 in normal peripheral blood was in all cases significantly lower than mobilised peripheral blood.

### Proportions of CD34^+ ^and/or CD133^+ ^cells co-expressing VEGFR2 in the different sources

Proportions of CD34^+ ^and/or CD133^+ ^cells which co-express VEGFR2 varied between sources. In CD34^+^-rich sources (i.e. excluding nPB), CB (8.93%) was the source with the highest percentage of CD34^+ ^cells expressing VEGFR2, compared to BM (5.43%), mPBp (3.92%) (Figure 3d). Although CB had the highest percentage of CD34^+^VEGFR2^+ ^cells, it was the HPC source with the lowest absolute number of CD34^+ ^cells and therefore showed no significant difference from BM and mPB samples in terms of numbers of CD34^+^VEGFR2^+ ^cells (Figure 3a).

The percentage of CD34^+ ^or CD133^+ ^cells co-expressing VEGFR2 was significantly higher in normal peripheral blood than in any other source tested (Figure 3d, e). However, due to the very low number of CD34^+ ^or CD133^+ ^cells in nPB, the total number of CD34^+ ^or CD133^+ ^cells co-expressing VEGFR2 was significantly lower than in CB, mPB or BM (Figure 3a, b).

Quantification of CD34^+^VEGFR2^+ ^cells as a proportion of the CD133^+ ^cells (CD34^+^VEGFR2^+^CD133^+^) was almost below limits of detection by flow cytometry when acquisition of a minimum of 50,000–100,000 leukocyte events was used. Apparently similar proportions of CD34^+^VEGFR2^+^CD133^+ ^cells were found between the different sources tested, with BM being the source with the lowest proportion compared to the other sources tested (Figure 3f).

### Frequencies of CFU-EPC in sources of HPC and in normal peripheral blood

**Figure 4:**
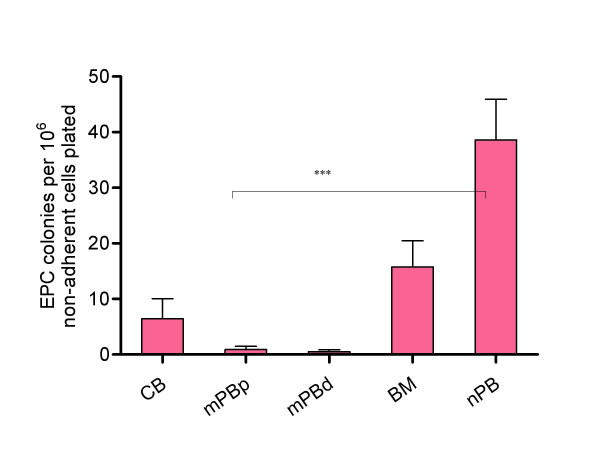
**Endothelial progenitor cell colony assay (CFU-EPC) in haematopoietic stem cell clinical transplant sources and normal blood**. Endothelial progenitor cell colonies (CFU-EPC) per 10^6 ^cells plated in the different sources tested. Normal peripheral blood (nPB)(n = 15), bone marrow (BM)(n = 7), cord blood (CB)(n = 11) and G-CSF-mobilised peripheral blood samples (mPBP, patient (n = 11); or mPBD, donor (n = 5)). (*** p < 0.001, Mann-Whitney test).

Normal peripheral blood, where the total numbers of CD34^+ ^and CD133^+ ^cells alone or co-expressing VEGFR2 are very low, gave the highest frequency of CFU-EPC (39.37 CFU-EPC/10^6 ^cells plated), (Figure 4). Thus, approximately 1 in 25,000 mononuclear cells (0.004%) in normal peripheral blood had the potential to proliferate and generate endothelial colonies, which is of the order of magnitude of HPC subpopulations found in normal peripheral blood. By contrast, the sources known to be rich in CD34^+ ^and/or CD133^+ ^(i.e. mPB, BM and CB) had much lower numbers of CFU-EPC. Of these sources, bone marrow gave the highest frequency of CFU-EPC, cord blood was lower, and G-CSF mobilised peripheral blood, the source with the highest number of cells expressing CD34 and/or CD133 jointly expressing VEGFR2, was virtually incapable of forming colonies. This was similar for both (autologous) patient (mPBp) or (allogeneic) healthy PBHPC donors (mPBd) (BM:15.7 CFU-EPC/10^6 ^cells plated; CB:6.4 CFU-EPC/10^6 ^cells plated; mPBp: 0.9 CFU-EPC/10^6 ^cells plated; mPBd: 0.6 CFU-EPC/10^6 ^cells plated) (Figure 3). Where CFU-EPC from bone marrow, cord blood or mobilised peripheral blood sources were seen they were morphologically different and much smaller than those generated from peripheral blood mononuclear cells. Individual cord blood cells also acquired spindle-shaped morphology under colony assay culture conditions while most of those from G-CSF mobilised samples remained rounded.

### Lack of correlation between any of the published immunophenotypic definitions of EPC progenitors and their ability to generate CFU-EPC

As well as the obvious contrast between the different sources in their content of putative EPC defined by CFU-EPC or defined by immunophenotypic assays, neither the proportions nor the absolute total numbers of subpopulations expressing CD34 or CD133 or VEGFR2 singly or in any joint combination showed any significant correlation with the number of endothelial progenitor cell colonies generated within or across any of the sources studied. The only trend that was found was between falling mean CFU-EPC numbers and falling mean CD14^+ ^cell proportions (of mononuclear cells) across normal peripheral blood, bone marrow and cord blood, respectively. This excluded G-CSF-mobilised peripheral blood whose mean CD14^+ ^cell proportions were similar to normal peripheral blood, but whose mean CFU-EPC numbers were least of all the groups. Neither mean nor individual values for CFU-EPC and CD14^+ ^proportions reached significance, but CD14 evaluation was introduced late in the study and only 5 individuals were evaluated in each category.

## Discussion

We have investigated the presence of currently proposed EPC phenotypes in the most common and practicable available sources of these cells for clinical use. All sources except normal peripheral blood (nPB) had high numbers of CD34^+ ^and CD133^+ ^cells which are generally regarded as correlates of haematopoietic progenitor cells (HPC), with mobilised peripheral blood (mPB) the source that had significantly higher total numbers of CD34^+ ^and CD133^+ ^by comparison to the other sources. mPB had 10 times more CD34^+ ^and CD133^+ ^cells/ml (205.8 and 146.6 × 10^3 ^cells/ml respectively) than normal peripheral blood. We found that the PBHPC were predominantly CD34^+ ^cells which co-expressed CD133, whereas these were in a minority in HPC in bone marrow or normal peripheral blood where singly expressing CD34^+ ^or CD133^+ ^were in the majority. These results agree very closely with that of De Wynter et al[44] who showed that 75% of CD34^+ ^from post-G-CSF apheresis samples were CD133^+ ^compared with 50% in CB and 35% in BM: normal peripheral blood was not studied. Results from mobilised peripheral blood samples for both patients donating PBHPC for autografts and healthy allogeneic PBHPC donors are similar, and indicates that these findings are not a reflection of underlying differences arising from any preceding therapy for patients' leukaemias. These phenomena relating to changes in HPC and EPC following G-CSF administration have been more extensively characterised by us (manuscript in preparation), and their underlying processes are under investigation.

It is currently believed that endothelial progenitor cells (EPC) are a subpopulation of CD34^+ ^and/or CD133^+ ^which express the more specific endothelial marker VEGFR2. In our study mobilised peripheral blood was the source found to have the highest number of CD34^+ ^and/or CD133^+ ^cells alone or in combination with VEGFR2 expression whereas normal peripheral blood was found to have the lowest numbers, mainly due to the low HPC numbers in normal peripheral blood. When proportions are considered, normal peripheral blood has the highest proportions of CD34^+ ^cells or CD133^+ ^cells which jointly express VEGFR2. However these overlap very little into cells expressing all three markers.

Since it has also been widely reported that EPC can be measured by an EPC colony assay (CFU-EPC) [29,30,32,33,35,45-49], this was also evaluated, to determine whether a phenotype-based or colony-based assay might be best for adoption by us for routine measurement of EPC in different clinical samples. Measurement of CFU-EPC can identify very low frequencies of such cells since individual colonies are obvious, and may provide a sensitive indicator of the capacity of mononuclear cells to produce endothelial cells. This assay has been used in a number of studies to measure CFU-EPC in different clinical situations and in healthy individuals for comparison since it was first described by Hill et al[27]. These studies measure CFU-EPC in normal peripheral blood as some means of assessing vasculogenic potency, and reduced numbers have been found in clinical conditions associated with ischaemic disease [27,50-54].

Since EPC have been associated with HPC, which are low in normal peripheral blood having less than one tenth of the numbers of CD34^+ ^and/or CD133^+ ^cells compared with the other sources, it was surprising to find that normal peripheral blood mononuclear cells was the source with the highest number of CFU-EPC. Of the HPC-rich sources, bone marrow was identified as the best source of CFU-EPC as compared to both cord blood and mobilised peripheral blood from both patients and allogeneic healthy donors, but never as high as normal peripheral blood. We have not found a correlation between numbers of CFU-EPC and the number of cells expressing CD34 and CD133 either alone or in combination, or in combination with expression of VEGFR2.

The contrast between normal peripheral blood having the greatest number of CFU-EPC and very few HPC, while mobilised PBHPC contain the most HPC but are virtually devoid of CFU-EPC, is striking. This would seem to indicate that CFU-EPC and EPC defined by immunophenotype are not the same subpopulations of mononuclear leukocytes in peripheral blood. Similar results were obtained by George et al[55], who suggest that while phenotype may provide numerical data, the CFU-EPC assay may provide some functional measure of their ability to proliferate. Others suggest that CFU-EPC are derived from CD14^+ ^monocyte-like cells [56-58], and while their contribution to vasculogenesis (and the relevance of the CFU-EPC assay) is disputed[59], it appears that there is a contribution to vasculogenesis by some CD14^+ ^cells[58,60-63]. This could explain why HPC-poor normal peripheral blood mononuclear cells can be as effective as bone marrow or enriched PBHPC in eliciting vasculogenesis in clinical or laboratory model ischaemia [37,64-66]: however, these appear to require *ex vivo *maturation or expansion to be effective. Our findings that the numbers of CFU-EPC in normal peripheral blood, bone marrow and cord blood reflect the proportions of CD14^+ ^cells in these mononuclear leukocyte sources may support this, but the fall that we find in peripheral blood CFU-EPC following administration of G-CSF appears to put PBHPC into a different category, perhaps reflecting the different CD34^+^/CD133^+ ^expression, and this is under further investigation by us.

Even within immunophenotypically defined subpopulations it is evident that the different sources differ in character, especially in whether CD34 and CD133 are expressed uniquely or jointly. If only one or other of these is assessed for co-expression of VEGFR2, it may be that for mobilised PBHPC that may encompass most VEGFR2-expressing HPC, whereas for normal peripheral blood or bone marrow a significant population of VEGFR2-expressing HPC would go undetected. The situation for cord blood appears to be intermediate. Most studies indicate that CD34^+^/CD133^+^/VEGFR2^+ ^cells can differentiate *in vitro *into cells expressing mature endothelial markers and that in general CD133 expression is lost during this process [67-70]. However, there are some studies which suggest that many HPC are plastic and that such markers can be up-regulated or down-regulated [10,16,18].

A number of recent clinical studies have employed autologous sources of haematopoietic stem cells to elicit apparent revascularisation of ischaemic tissues. However, there is no consistent definition of which cells are active in this revascularisation. If CFU-EPC reflects clinically relevant circulating EPCs it could be that peripheral blood mononuclear cells would be a better source of EPC for vascular repair/regeneration in a clinical setting and that G-CSF mobilised HPC might be the worst source of EPC. However laboratory models and clinical studies indicate that bone marrow or peripheral blood HPC selected by either CD34 or CD133 phenotype, or normal peripheral blood mononuclear cells matured *ex vivo *on fibronectin in "endothelial" cultures, are all effective in vasculogenesis. These EPC definitions need further clarification. We have undertaken overlapping complementary studies employing different cell subset enrichment and depletion which will expand on this (manuscript in preparation), and we continue to develop these in different models of vasculogenesis which may more directly reflect mature endothelial cell function.

## Conclusion

It has been proposed that EPC can be defined as cells expressing VEGFR2 (KDR) and one or both of the HPC markers CD34 and CD133. Cells expressing all three markers are very rare in any of the cell sources studied, but cells expressing VEGFR2 and either CD34 or CD133 are found and are most frequent in mobilised peripheral blood, following administration of G-CSF. That mobilised peripheral blood is the cell source with the lowest frequency of EPC as defined by CFU-EPC, while normal blood has the highest frequency of CFU-EPC yet by far the lowest frequency of any cells expressing HPC markers (CD34 or CD133), suggests that the phenotypically defined cells are distinct from the functionally defined CFU-EPC. Other relevant phenotypically defined characteristics, such as the degree of overlap of CD34 and CD133 expressing cells (joint expression of these HPC markers), differ in the different cell sources. There is no implication from the published clinical studies that any of these definitions of EPC reflects the actual vasculogenic capacity of a cell source, and the clinical evidence suggests that EPC are ubiquitous in peripheral blood and bone marrow and heterogeneous in character. Further studies are required to establish laboratory assays for EPC for measuring vasculogenic potency of clinical autograft cells that will be the equivalent of measuring CD34-expressing cells to assess haematopoietic potential. The relevance of the CFU-EPC assay is not established and requires definition of the cells which initiate EPC colony formation. The proposed phenotype assays are neither explicit nor comprehensive, and probably require more complex phenotype analysis or the identification of some exclusive marker which is a correlate of some directly measured aspect of vasculogenesis.

## Competing interests

The author(s) declare that they have no competing interests.

## Authors' contributions

GRB and OT conceived of the study, participated in its design, coordination and analysis, and drafted the manuscript. HR, JD and MLT authorized access to patient and blood donor samples for the study and contributed clinical expertise and oversight to the study design. OT carried out cell isolation, FACS analysis and colony assays as a component of her PhD thesis studies under the supervision of GRB. All authors read and approved the final manuscript.
